# Microscopic characteristics and sources of atmospheric dustfall in open-pit mining coal resource-based city in the arid desert area of Northwest China

**DOI:** 10.1038/s41598-024-56892-8

**Published:** 2024-03-15

**Authors:** Yayuan Deng, Hongxuan Wu, Tingning Zhao, Changqing Shi, Yan Zhang, Feng Li

**Affiliations:** 1 School of Soil and Water Conservation, Beijing Forestry University, Beijing, 100083 China; 2 Xifeng Water Authority, Guiyang, 551100 Guizhou Province China; 3 Wuhai Xinxing Coal Co., Ltd., Wuhai, 016000 Inner Mongolia Autonomous Region China

**Keywords:** Atmospheric science, Environmental sciences

## Abstract

Atmospheric dustfall is solid air pollutant, has a major impact on the environment and human health. The objective of this study was to investigate the microscopic characteristics and sources of atmospheric dustfall in open-pit mining coal resource-based city in the arid desert area of Northwest China. The characteristics of size and shape factors, variation of shape factors with size distribution, types of individual particles, and sources of atmospheric dustfall, which were collected in the open-pit mining area and surrounding areas, were analyzed by X-ray diffraction (XRD) and scanning electron microscopy coupled with an energy dispersive spectrometer (SEM–EDS) combined with graphical method and shape factors. The results showed that the atmospheric dustfall in all functional areas was dominated by coarse-grained particles. The shape of the atmospheric dustfall deviated from spherical shape, and with decreasing particle size, the difference in shape factors increased in each functional area. The EDS and XRD analyses indicated the presence of 13 types of particles. The sources were mainly local and included soil dust from each functional area; industrial dust, construction dust, biogenic impurities, fossil fuel combustion, wear products of motor vehicle parts, motor vehicle exhaust emissions, and emission and excreta from biological activities in each functional area except the desert area; emissions from a steel plant in the industrial area; coal-associated ore, coal dust, coal gangue emissions, and emissions from the spontaneous combustion of coal gangue in the open-pit mining area; secondary chemical crystallization products in the industrial area and the open-pit mining area; dust generated by vehicles abrading the surface of the off-mine coal road and in the open-pit mining area.

## Introduction

The arid and semi-arid regions in mainland China is considered to be one of the worst emission source areas of atmospheric particulate matter on Earth^1,2^. Especially in the coal resource-based cities, mining operations can bring economic benefits to the local region but also cause serious environmental pollution^3,4^. Atmospheric dustfall is the part of atmospheric particulate matter which can settle naturally^5–7^, it is considered to be an important indicator for evaluating atmospheric pollution, atmospheric dustfall not only affects climate and atmospheric visibility but also causes serious harm to human health, leading directly or indirectly to socio-economic losses^8–12^.

Particle size and morphology are typical microscopic characteristics of atmospheric dustfall, which can directly reflect the source and cause information^13,14^. Particle size has been studied extensively and is an essential part of microscopic characteristics. Morphology of atmospheric dustfall has been mainly based on the subjective description using the results of observed images^15^. Shape factor is used to quantitatively characterize the shape of particulate matter, which is mainly used in studies of soil, rock, tailings, and atmospheric fine particles etc^16–20^, but it is rarely used in the study of atmospheric dustfall. So using the shape factors to statistically analyze the morphological characteristic of atmospheric dustfall, can reflect objectively results. Moreover, these characteristics were normally studied separately. There is a lack of research on the correlation between the morphology and particle size distribution of atmospheric dustfall, so the correlation between these two microscopic characteristics is worth exploring.

The source analysis of atmospheric dustfall mainly utilizes receptor model methods, which are classified into chemical method and microscopic method^21–23^. Chemical method can be quantitatively analyzed pollution sources by analyzing their source spectra and contribution rates^23^. Microscopic method is used for the qualitative and semi-quantitative analyses of pollutants^24,25^. Compared with chemical method, microscopic method has certain subjectivity, and it does not allow for exact quantitative analysis. However, using microscopic method in conjunction with the current state of the study area, specific dust sources can be qualitatively analyzed.

Wuhai, an open-pit mining coal resource-based city, is located in the arid desert area of Northwest China, where has an extremely fragile ecosystem^26,27^. Previous researchs have investigated the temporospatial distribution of dust concentration and the characteristics of heavy metal pollution of atmospheric dustfall, as well as using principal component analysis to analyze the pollution sources and their contribution^28,29^. However, there have been no studies on microscopic characteristics of atmospheric dustfall and specific dust source analysis in Wuhai. In this study, atmospheric dustfall samples were collected during the windy period in 2018. The sizes, shapes, and elemental compositions of single particles were determined by SEM–EDS, and their mineral compositions were determined by XRD. The characteristics of size, shape, and variation of shape factors with size distribution were analyzed. The types of individual particles were determined based on the elemental and mineral composition, and the specific dust sources of atmospheric dustfall were then analyzed in relation to the characteristics of five functional areas. The results of this study can verify the correlation between microscopic characteristics of atmospheric dustfall and provide new ideas for the study of correlation among atmospheric particulate matter characteristics, and using shape factors can more objectively reflect the morphological characteristic. The identification of specific dust sources can improve the analysis of the source of atmospheric dustfall, provide theoretical support for conducting accurate dust control and a scientific basis for air pollution control measures in open-pit mining coal resource-based cities in the arid desert area of Northwest China.

## Materials and methods

### Study area

**Figure 1:**
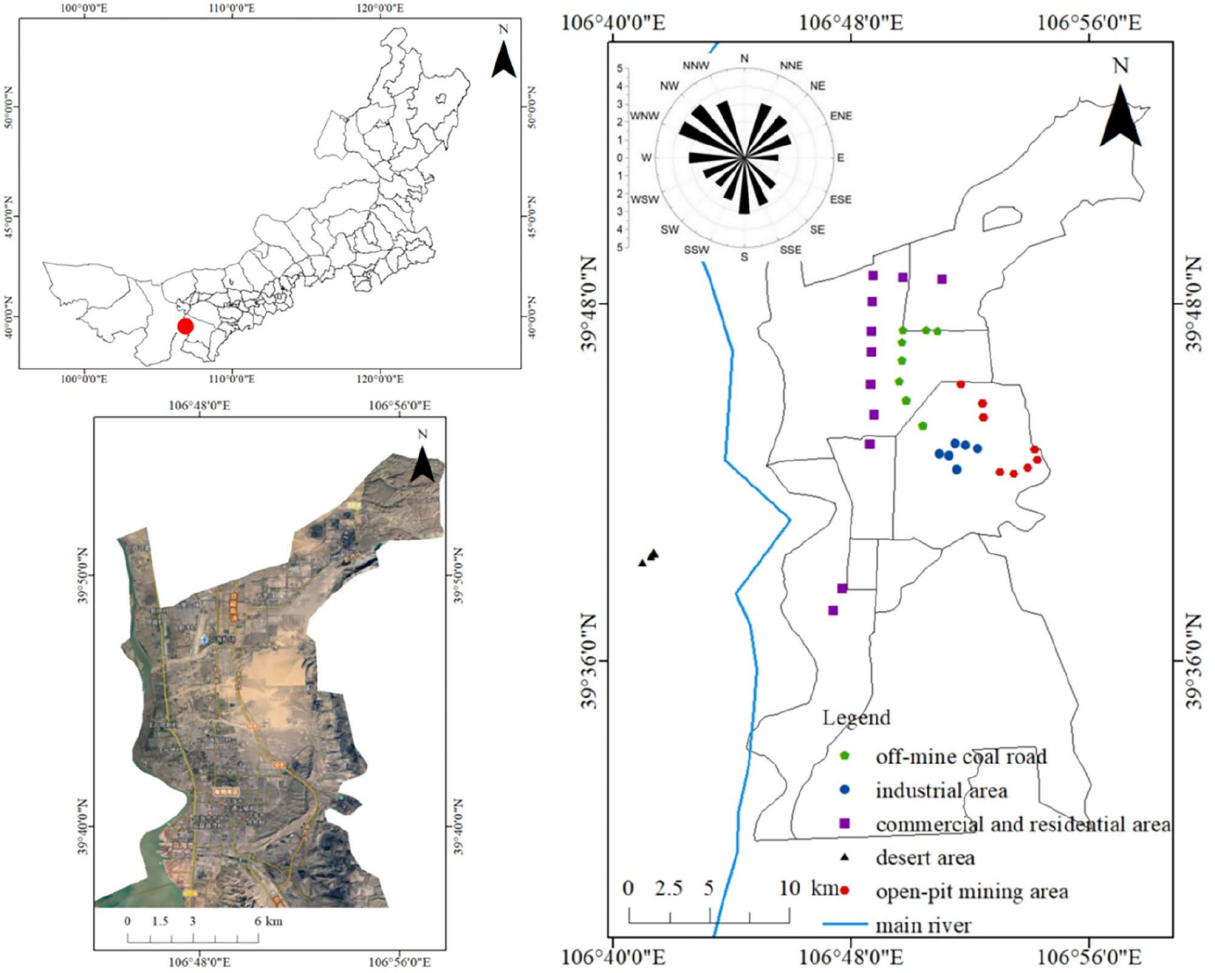
Image of sampling sites of atmospheric dustfall, and the wind regime.

Wuhai City is located in the arid desert area of Northwest China and the southwest of the Inner Mongolian Autonomous Region of China (Fig. 1, 106°52′50′′–106°54′17′′E, 39°42′03′′–39°42′36′′N). Since 2006, the coal mining method in this city has gradually changed from underground mining to open-pit mining. The study area is a continental monsoon climate area. The annual average wind speed is 2.9 m/s, the annual instantaneous maximum wind speed is 33 m/s, the annual average relative humidity is 42%, the annual average precipitation is 159.8 mm, and the annual evaporation is 3289 mm. Annual aeolian sand days are more than 80 days, and the days of strong wind are mainly concentrated in the windy period (March–May). The study period is the windy period (March–May) of 2018 with an average maximum wind speed of 7.15 m/s. Aeolian sand days occurred sixteen times, and the prevailing wind direction was northwest.

In this area, soil types can be mainly divided into six types, gray desert soil, brown soil, chestnut soil, sandy soil, meadow soil, and saline soil, among which gray desert soil and brown calcium soil are the main components. The vegetation type is mostly desert steppe vegetation, mainly herbs, shrubs, and small trees. The vegetation coverage of Wuhai City is 30.26% (including crops on agricultural land and green plants on construction land) during the study period.

The study area was divided into five functional areas, open-pit mining area, off-mine coal road, industrial area, commercial and residential area, and desert area (Table 1).

**Table 1:** Introduction of the five functional areas.

Functional zone	Location	Description	Position relationship involves wind direction
Open-pit mining area	In the Xinxing Coal Mine to the northeast of Wuhai City	Including the entrance and exit of the mine, mining pits, coal lines, coal waste dumps, coal gangue dumps, and office and living areas The coal gangue dumps were managed on a large scale from 2015 to 2016, but some spontaneous combustion still occurs The surface of coal lines is a clay-bound gravel surface	/
Off-mine coal road	In the northwest of the open-pit mining area	The road surface is asphalt concrete, managed with regular watering and dust suppression	Located upwind of the open-pit mining area and industrial area
Industrial area	In the west of the open-pit mining area	Accommodates steel plants, coal coking plants, and other industries	/
Commercial and residential area	In the mixed residential and commercial housing construction of the Haibowan district	/	Located upwind of the open-pit mining area, off-mine coal road and industrial area
Desert area	In the Ulaanbuhe Desert on the west bank of the Yellow River	Underlying surface is semi-fixed dune	Located to the west of the other four functional areas

### Sample collection

Samples were collected using deposition cylinders (cylindrical plexiglass cylinders) with reference to the Chinese national standard “GB/T15265-1994”, stating the following layout principles. To avoid damage to or replacement of the dust deposition cylinders by humans, the cylinders are usually placed on the top floor of low-level buildings or on electric poles; they should be located far away from tall buildings to avoid disturbance by unnatural factors; the cylinders should be placed at the same height, which was set at a height of 2 m above ground, to avoid the impact of dust from platforms.

In this study, the dust deposition cylinders were fixed on electric poles in open and flat areas without tall buildings. After the samples were collected, debris such as branches and leaves were removed, and the samples were stored in dry and sealed hermetic bags. Samples were collected from March 1, 2018, to May 31, 2018.

A total of 37 sampling points were set up in five functional areas (Fig. 1), including eight sampling points in the open-pit mining area, eight sampling points on the off-mine coal road, six sampling points in the industrial area, eleven sampling points in the commercial and residential area, and four sampling points in the desert area.

### Samples analysis

#### Scanning electron microscopy coupled energy dispersive spectrometer

Samples of each functional area were evenly mixed, and the resulting five mixed samples were analyzed as representatives of atmospheric dustfall in the five functional areas. Scanning electron microscopy coupled with an energy dispersive spectrometer (SEM–EDS) (Hitachi-SU8010, Japan) was used for the determination of particle size, shape observations, and element analyses of single particles.

For each functional area, taking an appropriate amount of each sample and evenly dispersing it onto 5 conductive adhesives (conductive adhesive size: 2 × 2 mm) pasted on the aluminum mount, and a very thin film of gold was deposited on the sample surface before testing. The FESEM images of particles were acquired at magnifications of 200, 500, 800, 1200, and 2000 by using the five-point sampling method. Ten images were acquired for each sample with a total number of particles between 900 and 1300 per sample. The SEM is used to select the single particles, and the equipped EDS is used to scan them, so that the energy spectra of the main elements and the proportion of the content of individual elements can be obtained.

The SEM–EDS system was equipped with a cold field-emission electron gun, operated under vacuum with a voltage of 3 kV and a current of 10 μA; the EDS was set at an accelerating voltage of 15 kV, a fixed angle of 35°, and an elemental analysis range of _4_Be–_92_U.

Image-Pro Plus 6.0 software can determine the number of particles and various particle-parameters in SEM images, applicable to the research of correlation between particle size distribution and shape factors of atmospheric dustfall in this study. So after collecting the FESEM images, importing them into this software for particle size analysis and shape factor parameter acquisition.

### X-ray diffraction

XRD is a non-destructive technique used to identify mineral phases and is well established in the determination of the mineral compositions of powders. This method is the most commonly applied technique for determining the mineral content of dust. It can be used for qualitative and semi-quantitative analysis of mineral compositions, but it is not possible to accurately quantify them, so in this study it was only used to qualitatively analyze.

In this study, atmospheric dustfall was examined using the XRD instrument (MiniFlex 600, Rigaku, Japan). The samples were thoroughly ground using a mortar and pestle, and then were pasted onto slides and placed on an XRD sample stage to measure with Cu Kα radiation (40 kV and 15 mA) in the 2θ range of 3° to 50° with a scanning rate of 0.05°/min and a step size of 0.01° in order to get the XRD spectra. Then compared the XRD spectra with the standard card data by Jade 6.5 to determine the mineral phases.

### Calculation methods

#### Graphical method

In this study, a graphical method was chosen to analyze the particle size of atmospheric dustfall. The characteristic parameters of the grain size include the mean diameter (*M*_Z_), standard deviation (*S*), skewness (*S*_k_) and kurtosis (*K*_G_), which were calculated by using the Folk-Ward formula^30^ (Table 2), where D_5_, D_16_, D_25_, D_50_, D_75_, D_84_, and D_95_ indicate the particle size corresponding to 5%, 16%, 25%, 50%, 75%, 84%, and 95% of the cumulative percentage, respectively.

**Table 2:** Formulas of grain-size parameters.

Grain-size parameter	Folk–Ward formula
*M*_Z_ (mean diameter)	${(D}_{16}+{D}_{50}+{D}_{84})/3$
*S* (standard deviation)	${(D}_{84}-{D}_{16})/4+{(D}_{95}-{D}_{5})/6.6$
*S*_k_ (skewness)	$({D}_{84}+{D}_{16}-2{D}_{50})/2({D}_{84}-D16)+({D}_{95}+{D}_{5}-2{D}_{50})/2({D}_{95}-{D}_{5})$
*K*_G_ (kurtosis)	$({D}_{95}-{D}_{5})/2.44({D}_{75}-{D}_{25})$

#### Shape factors

FESEM images were analyzed with Image-Pro Plus 6.0 software to obtain primary and secondary parameters of the particles. Roundness was directly derived from the software, and Aspect ratio, Convexity and roughness were calculated by the respective formulas. The shape factors chosen in this study were two-dimensional parameters.

Roundness (*R*) is defined as the similarity of a particle to a circle,

1 $$ {\text{R}} = {\text{P}}^{2} /4{\pi A} $$

where *P* is the projected perimeter, and *A* is the projected area. The range of *R* is [1,∞]. A perfectly spherical particle shape has an *R* value of one.

Aspect ratio (*AR*) is defined as the length of a particle shape^17,31^,

2 $$ {\text{AR}} = {\text{F}}_{{{\text{min}}}} /{\text{F}}_{{{\text{max}}}} $$

where *F*_max_ and *F*_min_ are the maximum and minimum Feret diameters. The range of *AR* is [0,1]. When *AR* is close to one, the particle shape is close to a sphere or square. When A approaches zero, the particle shape becomes column- or flake-like.

Convexity (*Con*) is defined as the angularity of a particle shape^17,31^,

3 $$ {\text{Con}} = {\pi D}_{{{\text{Aeq}}}} /{\text{P}} $$

where *D*_Aeq_ is the particle equivalent diameter. The range of *Con* is [0,1]. When *Con* is close to one, the angles of the particles are sharp; otherwise, the angles are blunt.

Roughness (*r*) is defined as the undulation of a particle shape^32^,

4 $$ {\text{r}} = ({\text{P}}/{\text{P}}_{{\text{C}}} )^{2} $$

where *P*_C_ is the circumscribed polygon perimeter of the particle. The standard value of *r* is one, and the closer *r* is to one, the smoother is the particle surface, and conversely, the rougher is the surface.

## Results and discussion

### Particle size characteristics of atmospheric dustfall

In this study, the widely used Udden-Wentworth classification method was chosen for particle size classification^33,34^, and the particle size was converted to the Φ value by using the equation Φ = − log_2_D proposed by Krumbein^35^. The particles were classified as clay (Φ > 9), very fine silt (8–9Φ), fine silt (7–8Φ), medium silt (6–7Φ), coarse silt (5–6Φ), very coarse silt (4–5Φ), very fine sand (3–4Φ), fine sand (2–3Φ), medium sand (1–2Φ), and coarse sand (Φ < 1).

**Figure 2:**
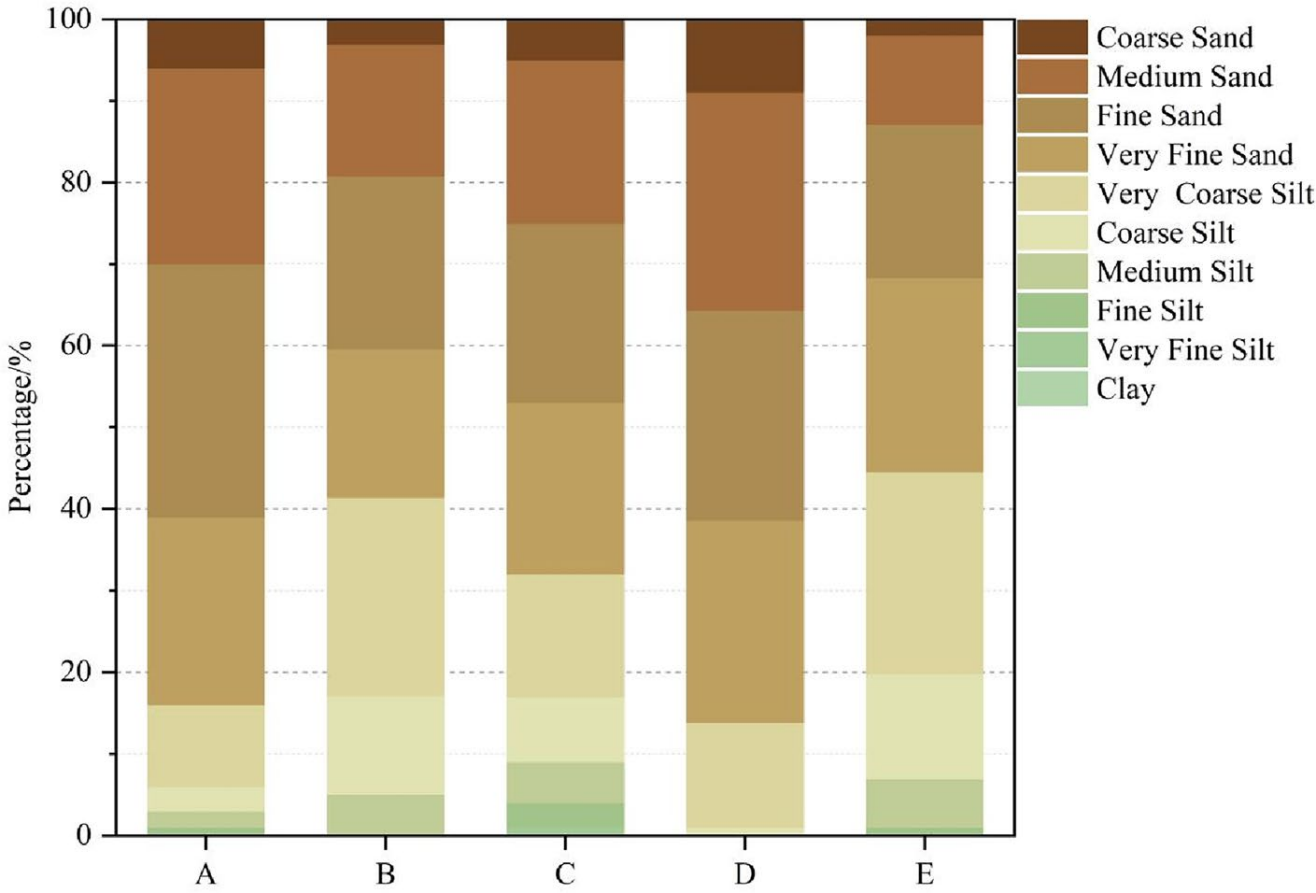
The cumulative percentage of particle size of atmospheric dustfall in each area (A—off-mine coal road; B—industrial area; C—commercial and residential area; D—desert area; E—open-pit mining area).

The results showed that the particle size distribution of atmospheric dustfall was in the Φ range of 0–8 in the open-pit mining area, the industrial area and on the off-mine coal road, was in the Φ range of 0–9 in the commercial and residential area, and was only in the Φ range of 0–5 in the desert area. Figure 2 presents the cumulative percentage of particle size of atmospheric dustfall in different functional areas. Considering a fraction of more than 10% the main distribution size, the particle sizes of atmospheric dustfall in the open-pit mining area and industrial area were mainly distributed in the ranges of 1–6Φ. For the off-mine coal road, the commercial and residential area, and the desert area, the main particle size distribution ranges were 1–5Φ.

**Figure 3:**
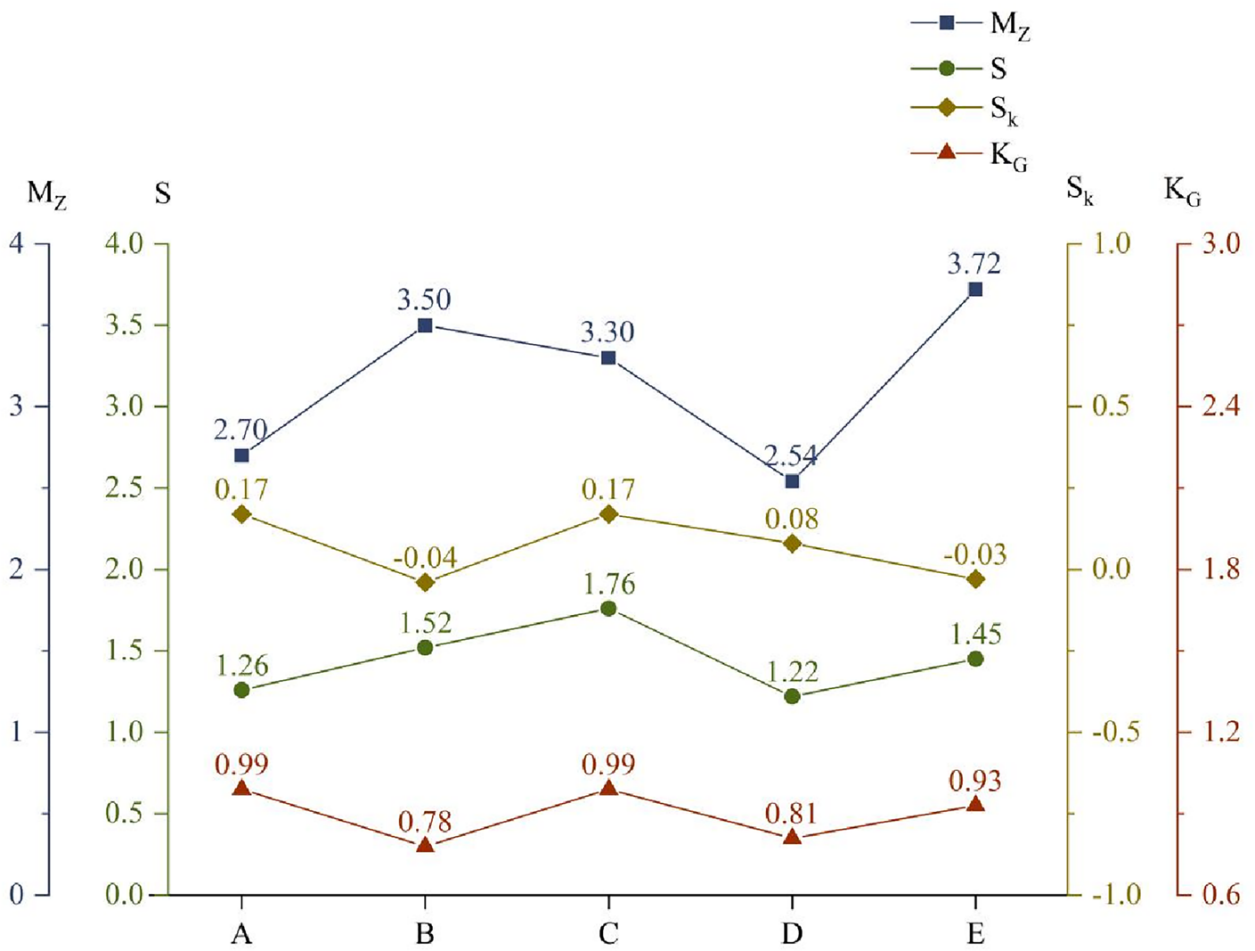
Grain-size parameters for atmospheric dustfall in each area (A—off-mine coal road; B—industrial area; C—commercial and residential area; D—desert area; E—open-pit mining area).

The grain-size characteristic parameters of atmospheric dustfall determined in each functional area are presented in Fig. 3. The maximum *M*_Z_ was found in the open-pit mining area, and the minimum *M*_Z_ was in the desert area. The values of *S* ranged from 1.22 to 1.76 and can be classified as poorly sorted in each functional area, as highlighted in Table 3. The values of *S*_k_ ranged from − 0.04 to 0.17, and can be classified as positive skewed on the off-mine coal road and in the commercial and residential area, and symmetrical in the industrial area, the desert area, and the open-pit mining area. The values of *K*_G_ ranged from 0.78 to 0.99, and can be classified as mesokurtic on the off-mine coal road, in the commercial and residential area, and the open-pit mining area, and platykurtic in the industrial area and the desert area.

**Table 3:** Grain-size parameter ranges and classifications.

Standard deviation(*S*)	Value	Skewness (*S*k)	Value	Kurtosis (*K*_G_)	Value
Very well sorted	< 0.35	Very negative skewed	− 0.3 to − 1.0	Very platykurtic	< 0.67
Well sorted	0.35–0.50	Negative skewed	− 0.1 to − 0.3	Platykurtic	0.67–0.90
Moderately well sorted	0.50–0.70	Symmetrical	− 0.1 to 0.1	Mesokurtic	0.90–1.11
Moderately sorted	0.70–1.00	Positive skewed	0.1–0.3	Leptokurtic	1.11–1.50
Poorly sorted	1.00–2.00	Very positive skewed	0.3–1.0	Very leptokurtic	1.50–3.00
Very poorly sorted	2.00–4.00			Extremely leptokurtic	> 3.00
Extremely poorly sorted	> 4.00				

Combining the size distribution characteristics and particle size characteristics, the proportion of atmospheric dustfall with sizes larger than 31 μm was greater than 90% in each functional area. The atmospheric dustfall in all functional areas was dominated by coarse-grained particles, which the highest content was very coarse silt in the industrial area and the open-pit mining area and fine sand on other three functional areas. It has been previously reported that when the wind speed is greater than or equal to the threshold wind velocity, the transport distance was determined by the particle size, and different particle sizes can only be transported over long distances under different dynamic conditions. Particles greater than 10 μm less than 100 km, and particles exceeding 31 μm can generally be transported less than 1000 m^36^. Consequently, the atmospheric dustfall in each area was mainly near-source material, which was highly influenced by local sources.

Previous reports showed that the atmospheric dustfall is mainly composed of fine silt, coarse silt and fine sand in underground coal mining resource-based city in the arid desert area of Northwest China^37^, which is similar to the results of this study. In China’s economically developed cities, it is mainly composed of the particles smaller than very coarse silt^38,39^. It can be seen that the main particle size distribution range of atmospheric dustfall in coal resource-based cities is larger than that in economically developed cities. The industrial activities, coal mining activities, and spring wind activities have a significant effect on the particle size distribution of atmospheric dustfall in coal resource-based cities in the arid desert area of northwest China.

### Shape factors of atmospheric dustfall

Shape factors are generally used to describe the degree of deviation of particulate matter from spherical particles. Since particle shapes are difficult to quantify, software-based image processing and morphological analysis is an effective approach for characterizing the shape and size distribution of particles. In this study, the shape information of individual particles was extracted from two-dimensional SEM images using Image-Pro Plus 6.0 software, and the shape characteristics of atmospheric dustfall in each functional area were obtained according to the shape factors.

Table 4 provides the mean values and standard deviations of the different shape factors of atmospheric dustfall in the selected five functional areas of Wuhai. The Roundness value in the desert area was closest to one with minimal variations, while being most deviated from one in the industrial area with maximal variations, the variation in each functional area was in the range of 0.04–0.10. Aspect ratio was closest to one in the desert area and deviated from one to the largest extent in the commercial and residential area, the variation in each functional area was in the range of 0.02–0.03, and the overall variation was small. Convexity was closest to one in the desert area and exhibited their greatest deviation from one in the industrial area. The variation in each functional area was between 0.01 to 0.03, and the overall variation was small. Regarding roughness, the mean value and overall distribution of atmospheric dustfall were similar in each functional area with no significant differences.

**Table 4:** Shape factor values of each area (Values = mean ± standard deviation).

Functional area	Roundness	Aspect ratio	Convexity	Roughness
Off-mine coal road	1.37 ± 0.10	0.65 ± 0.02	0.87 ± 0.02	1.04 ± 0.01
Industrial area	1.40 ± 0.10	0.64 ± 0.03	0.86 ± 0.03	1.05 ± 0.02
Commercial and residential area	1.37 ± 0.04	0.63 ± 0.03	0.87 ± 0.01	1.05 ± 0.01
Desert area	1.33 ± 0.04	0.66 ± 0.02	0.88 ± 0.01	1.04 ± 0.01
Open-pit mining area	1.35 ± 0.06	0.65 ± 0.02	0.87 ± 0.02	1.05 ± 0.01

There was no significant difference in roughness of atmospheric dustfall among the five functional areas, indicating that this factor was less affected by the different functional areas. Except for the desert area, the differences in Roundness, Aspect ratio and Convexity of atmospheric dustfall were small in each functional area, which were found to be mainly influenced by the dominant wind direction and the proximity of each functional area. The particle shape of atmospheric dustfall was closest to spherical shape in the desert area and deviated most from spherical shape in the industrial area and the commercial and residential area, which indicated that the angles of particles were sharper in the areas influenced by human factors. In general, the shape of atmospheric dustfall deviated from spherical shape in each functional area, which indicated that the impact of wear by atmospheric transportation was small, and the dustfall was not transported over long distances but was mainly influenced by local sources.

### The correlation between particle size distribution and shapes of atmospheric dustfall

**Figure 4:**
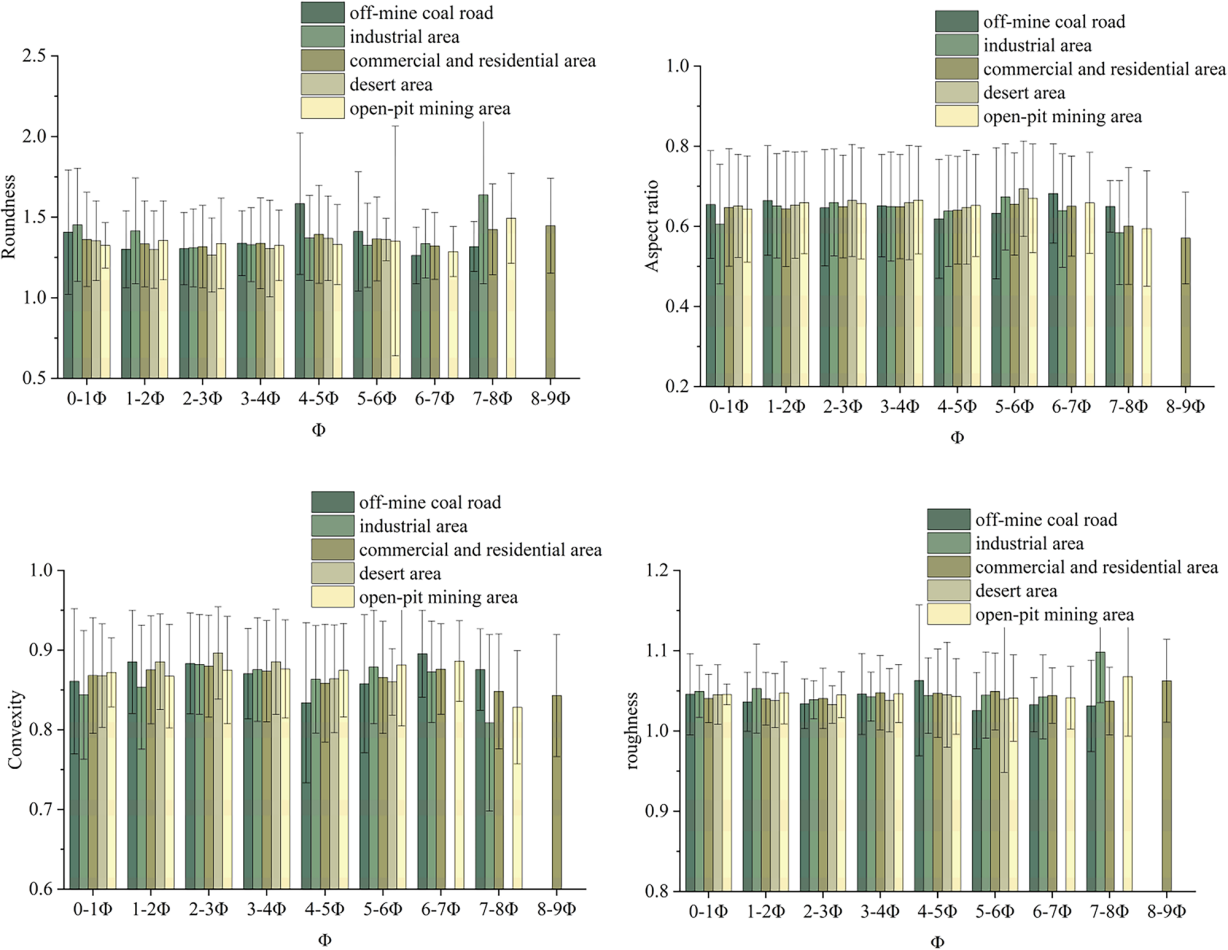
Shape factor values of atmospheric dustfall for different size distribution ranges in each functional area.

Figure 4 depicts the variation of shape factors with size distribution in the five functional areas. Except for the desert area, the values of Roundness and roughness showed a W-shaped variation with increasing particle size (Φ), while the values of Aspect ratio and Convexity exhibited an M-shaped trend. With increasing Roundness values, roughness increased and Aspect ratio and Convexity decreased, indicating that a particle shape was closer to a circle and its surface was smoother. Except for the off-mine coal road, the maximum values of Roundness and roughness and the minimum values of Aspect ratio and Convexity of atmospheric dustfall in each functional area were at the maximum Φ value.

The variations of shape factors with size distribution in each functional area were similar. With increasing Φ, the difference in the shape factor values among the functional areas increased, indicating a greater influence of the regional difference with decreasing particle size. Previous studies did not report on the relationship between the shape variation of atmospheric dustfall and particle size in different functional areas. The results of this study showed that the difference in the shape of atmospheric dustfall in different functional areas was significantly correlated with the particle size distribution. So the microscopic characteristics do not exist independently but affect each other, the correlation between them needs to be further investigated.

### Mineral composition of atmospheric dustfall

**Figure 5:**
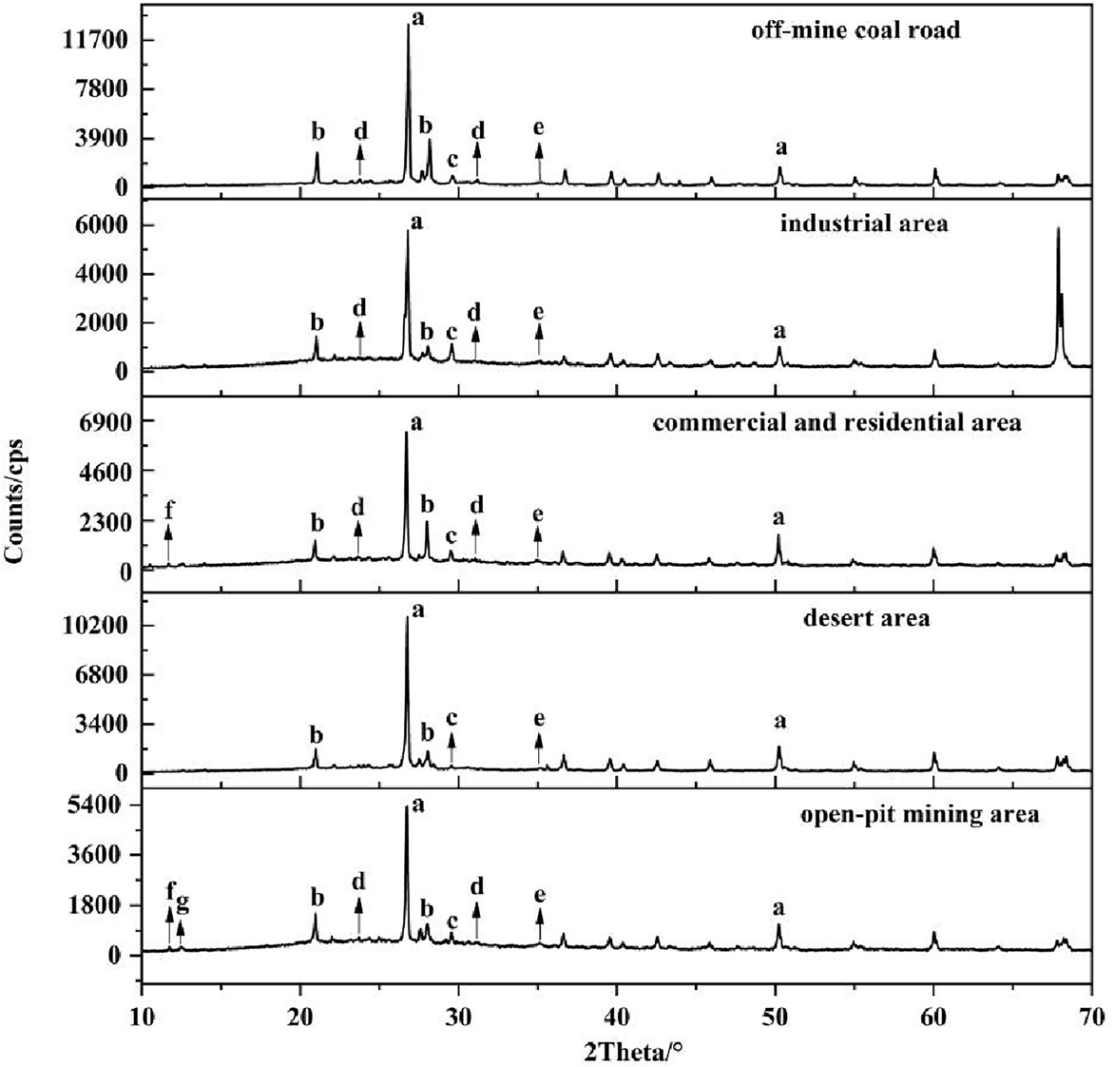
XRD determination of the mineral composition of atmospheric dustfall from different areas (a—quartz; b—feldspars; c—calcite/CaCO_3_; d—dolomite; e—muscovite; f—gypsum; g—kaolinite).

XRD data of the atmospheric dustfall from the five functional areas were acquired, and the minerals present in the samples were determined by comparing the characteristic XRD peaks with the RRUFF database of the reference standards. The compositions in each functional area were found to be similar with only partial differences shown in Fig. 5. The most obvious strong peaks in the XRD patterns of the atmospheric dustfall of each functional area in this study were attributed to quartz (a). Feldspar (b), calcite (c) and muscovite (e) were also detected in all functional areas. Dolomite (d) was detected in all functional areas except the desert area, gypsum (f) was detected in the commercial and residential area and the open-pit mining area, and kaolinite (g) was only detected in the open-pit mining area. In addition, non-crystalline particulate matter was not detected by XRD^2^. The low content of particulate matter was also one of the reasons why they could not be detected by this technique.

XRD is capable of analyzing crystalline mineral particles in the physical phase, effectively resolving the mineral composition of atmospheric particles, but is unable to identify non-mineral particles, whereas EDS is used to identify particle types by determining the elemental composition of particulate matter, and can identify non-mineral particles in atmospheric dustfall^40^. Therefore, the determination of the compositions of atmospheric dustfall by XRD could be complemented by EDS to identify other particle types that were not detected by XRD.

### Elemental composition of atmospheric dustfall

**Figure 6:**
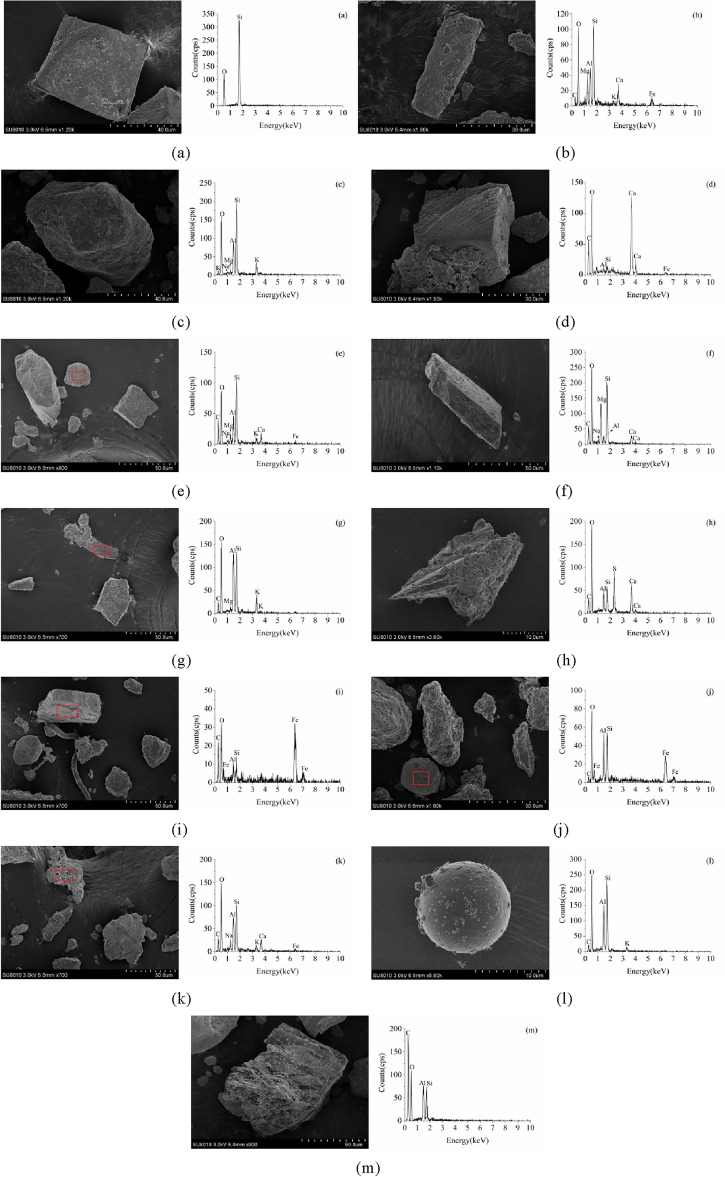
EDS spectra of different types of particulate matter.

Different types of single particles retrieved from the five functional areas were analyzed. Based on the EDS results, a total of 13 particle types were identified, including quartz, feldspar (plagioclase and potassium feldspar), calcite, kaolinite, dolomite, muscovite, gypsum, Fe-rich mineral, Fe-rich particles, soot aggregate, fly ash, and biogenic impurities (Fig. 6).

Quartz is displayed in Fig. 6a and constituted a bulk particle with sharp edges, an elemental [Si + O] content greater than 99%, and small amounts of other crustal elements. Figure 6b and c present feldspar. The columnar particle in Fig. 6b was identified as plagioclase, which surface was covered with crystalline impurities, and its main elemental content decreased in the order O > Si > Fe > Al > Ca > C > Mg. Figure 6c features a granular particle of potassium feldspar with a main elemental content decreasing in the order O > Si > Al > K > Mg. The elements O, Si, and Al are the main elements of feldspar with an Al:Si ratio of 1:3, and the most common feldspars are included in the KAlSi_3_O_8_–NaAlSi_3_O_8_–CaAl_2_Si_2_O_8_ system. Figure 6d shows a square particle of calcite, and its main elemental content followed the order O > Ca > C > Si > Al. A granular particle of kaolinite with porous surface is displayed in Fig. 6e. The content of the main elements of this particle decreased in the order O > C > Si > Al > Fe > Ca > Mg. Figure 6f presents dolomite as an irregular particle with a main elemental content of O > C > Ca > Mg > S > Si > Al and a Ca:Mg mass ratio of 5:3, corresponding to the chemical formula of dolomite, CaMg(CO_3_)_2_. An irregular particle of muscovite, which surface was covered with crystalline impurities, is displayed in Fig. 6g, the main elemental content was O > Si > Al > C > K > Fe, and the chemical formula of muscovite, KAl_2_(AlSi_3_O_10_)(OH)_2_, corresponded to the found elemental mass ratio. Figure 6h shows an irregular and sharp-edged particle of gypsum, and the main elemental content decreased in the order O > Ca > S > C > Si > Al, gypsum has a Ca:S mass ratio of 5:4, and the established elemental mass ratio of the particle agreed well with the theoretical value. Figure 6i and j show Fe-rich particles, an irregular particle in (i) with a main elemental content of Fe > O > C and an Fe content of 63.3% and a subspherical particle in (j) with a main elemental content of Fe > O > Si > Al > C and an Fe content of 51.1%. The particle in Fig. 6k was soot aggregate, usually appearing in the form of chains and fluff^41,42^, having the main elemental content of O > Si > C > Al > Ca > Fe > K in decreasing order. A square particle of fly ash is displayed in Fig. 6l. The main elemental content in this particle followed the order O > Si > Al > C > K. This type of particle is mainly formed at high temperatures^43^, and the surface is smooth and mostly aggregated with other particles^6^. Figure 6m shows biogenic impurity as an irregular particle with porous surface with a main elemental content of C > O > Si > Al. In previous studies, particulate matter with a [C + O] content greater than 75% was defined as biogenic particles; the [C + O] content detected in this study exceeded 80%.

### Main sources of atmospheric dustfall

Combining the results of mineral composition and elemental composition analysis, 13 types of particles were identified and classified into five categories: silicate particles, carbonate particles, sulfate particles, Fe-rich particles, and other particles.

#### Silicate particles

Silicate particles are mainly composed of O, Si, and Al and contain small amounts of K, Ca, Mg, Na, or other elements. In this study, quartz, feldspar, kaolinite and muscovite belonged to the category of silicate particles, and all were found in the five functional areas. Silicate particles are crustal components widely distributed in nature^44^. Those observed in this study had significantly sharp edges, indicating that they had not been transported over long distances and originated mainly from local soil dust^7,45^, of which kaolinite was mainly produced by weathering of silicate particles. Except for the desert area, silicate particles in each functional area might also stem from industrial dust and construction dust and might be affected by human factors^14^. A large amount of coal dust is generated during the coal mining process in the open-pit mining area, and the main constituent minerals of coal dust include kaolinite, quartz, and gypsum^46^. Therefore, coal dust was one of the sources of kaolinite, quartz, and gypsum in this study. Kaolinite from coal-associated ore was also a source of kaolinite in atmospheric dustfall^47^.

#### Carbonate particles

Carbonate particles are mainly composed of carbonate anions (CO_3_)^2−^ and metal cations such as Ca^2+^, K^+^, Mg^2+^ and Na^+^. In this study, calcite and dolomite belonged to the category of carbonate particles, which were found in the five functional areas. Carbonate particles are crustal components and are widespread in nature. The observed features were similar to those of the silicate particles, indicating that they had not been transported over long distances and mainly originated from local soil dust. Except for the desert area, carbonate particles in each functional area might also stem from industrial dust and construction dust. Large coal transportation vehicles using the concrete road caused the abrasion of the road surface and the production of calcite. These particles were affected by the wind and driven into the atmosphere by the movement of vehicles to be one source of calcite on the off-mine coal road.

#### Sulfate particles

Sulfate particles are mainly composed of sulfate anions (SO_4_)^2−^ and metal cations. In this study, gypsum belonged to the category of sulfate particles and was found in the industrial area, the commercial and residential area and the open-pit mining area. The presence of sharp edges in the gypsum indicated that it mainly originated from a local emission source. Gypsum is a widely used industrial and construction material. In this study, gypsum mainly originated from industrial dust and construction dust caused by human factors. In addition to the coal dust generated during the coal mining operations, the chemical reaction of calcite (CaCO_3_) present in the atmosphere and harmful sulfur dioxide, generated from the spontaneous combustion of coal gangue in the open-pit mining area and from the coal coking plant in the industrial area, would also generate gypsum, indicating that a part of gypsum was the secondary crystallization product of the chemical reaction between atmospheric particulate matter and acid gases^48,49^.

#### Fe-rich particles

Fe-rich particles were of subspherical and irregular shapes with Fe contents greater than 50%. Subspherical Fe-rich particles were mainly stemmed from wear of Fe-containing parts of motor vehicles^25,50^ in all functional areas except the desert area and were also emitted from steel plants in the industrial area. Irregular Fe-rich particles were present in all functional areas, mainly originating from local soil dust^51^, but the coal gangue emission in the open-pit mining area was also a source of these particles^52^.

#### Other particles

Soot aggregate and fly ash were present in all functional areas except the desert area. Soot aggregates were attributed to vehicle exhaust emissions from gasoline and diesel combustion and the incomplete combustion of biomass and fossil fuels^53,54^. Fly ash originated from the full combustion of biomass and fossil fuels, as well as the spontaneous combustion of coal gangue during coal mining operations^55,56^ in the open-pit mining area. Biological impurities were present in all functional areas, and based on previous researches, they might stem from biological excreta, plant debris, pollen and spores, as well as human hair and dander^7^.

Except for the desert area, the other four functional areas are located in the northeast of Wuhai City on the east bank of the Yellow River. The study period was in the windy period, which has the most days of high wind activity over the year, and the prevailing wind direction was northwest. Due to the prevailing wind direction, the other four functional areas were less influenced by the desert area as the source of atmospheric dustfall. Sources of silicate particles, carbonate particles, Fe-rich particles, and other particles in atmospheric dustfall in the industrial area and the open-pit mining area were influenced by the commercial and residential area and the off-mine coal road. The sources of atmospheric dustfall from these areas were mainly affected by human factors. Therefore, it is necessary to focus on road surfaces, industrial emissions, and coal mining operations to take reasonable and effective dust suppression measures to reduce the impact of atmospheric dustfall in Wuhai and other cities located downwind of the air pollution.

## Conclusion

1. The atmospheric dustfall was mainly composed of coarse silt, very coarse silt, very fine sand, fine sand, and medium sand in the industrial area and the open-pit mining area. In the commercial and residential area, the desert area, and on the off-mine coal road, the atmospheric dustfall mainly contained very coarse silt, very fine sand, fine sand, and medium sand. The atmospheric dustfall in all functional areas was dominated by coarse-grained particles.
2. The shape of the atmospheric dustfall deviated from spherical shape in all functional areas. There was no significant difference in roughness of the atmospheric dustfall in the five functional areas, indicating that it was less affected by the different functional areas. Except for the desert area, the differences in Roundness, Aspect ratio and Convexity of the atmospheric dustfall in each functional area were small and were found to be mainly influenced by the dominant wind direction and the proximity of each functional area.
3. The variation of shape factors with size distribution was similar in each functional area, and with decreasing particle size, the difference in shape factors increased in each area. The study on the microscopic characteristics of atmospheric dustfall can be further deepened from the correlation in the future.
4. The EDS and XRD analysis indicated the presence of quartz, feldspar, kaolinite, muscovite, calcite, dolomite, Fe-rich mineral and biogenic impurities in each functional area, while gypsum was present in the commercial and residential area, the industrial area and the open-pit mining area. Fe-rich particles, soot aggregates and fly ash was present in each functional area except the desert area.
5. The atmospheric dustfall in each functional area was not transported over long distances and was mainly influenced by local sources. Source analysis was conducted for the different types of particles of atmospheric dustfall, and the specific dust sources in each functional area were analyzed, which indicated that the main sources of atmospheric dustfall were soil dust in all functional areas. And other 15 sources were identified but were not existed in every functional area, because of their different specific functions and development priorities. But there is limitation to the source research in this paper. While the method provided qualitative analysis of source types, it did not allow for quantitative analysis of them.

## Data Availability

The data supporting the fndings of this article is included within the article.
